# The Role of the CALLY Index in 30-Day Mortality Prediction for Acute Mesenteric Ischemia: A Retrospective Cohort Study

**DOI:** 10.3390/medicina62010167

**Published:** 2026-01-14

**Authors:** Yeliz Simsek, Akkan Avci, Ahmet Burak Urfalioglu, Erdem Aksay, Adnan Kuvvetli, Ramazan Guven, Begum Seyda Avci, Saliha Dilek Oztoprak Hacioglu, Mustafa Oguz Tugcan

**Affiliations:** 1 Department of Emergency Medicine, Adana City Research and Training Hospital, Health Science University, 01230 Adana, Turkey; akkan.avci@saglik.gov.tr (A.A.); ahmet.urfalioglu@saglik.gov.tr (A.B.U.); erdem.aksay@saglik.gov.tr (E.A.); 2 Department of General Surgery, Adana City Training and Research Hospital, Health Sciences University, 01230 Adana, Turkey; adnan.kuvvetli@saglik.gov.tr; 3 Department of Emergency Medicine, Faculty of Medicine, Atlas University Hospital, 34203 Istanbul, Turkey; ramazan.guven@atlas.edu.tr; 4 Department of Internal Medicine, Adana City Training and Research Hospital, Health Sciences University, 01230 Adana, Turkey; begumseyda.avci@saglik.gov.tr; 5 Department of Public Health, Adana City Training and Research Hospital, Health Sciences University, 01230 Adana, Turkey; s.oztoprakhacioglu@saglik.gov.tr; 6 Department of Emergency Medicine, Faculty of Medicine, Cukurova University, 01330 Adana, Turkey; oguztugcan@cu.edu.tr

**Keywords:** acute mesenteric ischemia, CALLY, mortality

## Abstract

*Background and Objectives:* Acute mesenteric ischemia (AMI) lacks reliable prognostic biomarkers, and the prognostic performance of the C-reactive protein–albumin–lymphocyte (CALLY) index in this population has not been previously evaluated. This study aimed to assess the predictive value of the CALLY index for 30-day mortality in patients presenting to the emergency department (ED) with AMI. *Materials and Methods:* This retrospective cohort study included patients aged ≥18 years who presented to the ED with AMI over a 4-year period. Demographic and clinical data were collected. The CALLY index, neutrophil-to-lymphocyte ratio (NLR), platelet-to-lymphocyte ratio (PLR), and CRP-to-lactate ratio were calculated. The primary outcome was 30-day mortality. Univariate and multivariate logistic regression analyses were performed to identify predictors of mortality. A receiver operating characteristic (ROC) curve analysis was used to assess predictive performance. A *p*-value < 0.05 was considered statistically significant. *Results*: A total of 111 patients were included (mean age, 69.2 ± 11.8 years; 52.3% male). The most common comorbidities were hypertension and coronary artery disease (45% each). The 30-day mortality rate was 55.9%. In a univariate analysis, lower CALLY index values were associated with higher mortality (*p* = 0.011). However, the CALLY index did not remain independently associated with mortality in multivariate logistic regression analysis (*p* = 0.773). The ROC analysis indicated that the CALLY index had a modest but statistically significant ability to predict 30-day mortality (AUC = 0.64, 95% CI: 0.54–0.74, *p* = 0.011). At a cut-off value of 0.0015, the CALLY index showed a sensitivity of 55% and a specificity of 77%. *Conclusions*: The CALLY index had modest predictive value for 30-day mortality in patients with AMI.

## 1. Introduction

Acute mesenteric ischemia (AMI) is a severe clinical condition with a high mortality caused by insufficient blood flow to the small and large intestines. Although it accounts for less than 1% of all causes of acute abdomen in the emergency department (ED), it is a life-threatening medical emergency requiring urgent intervention [1,2]. Etiologically, AMI is classified into two main groups: primary and secondary. Primary AMI causes are classified, in order of frequency, as mesenteric arterial occlusion (MAO), non-occlusive mesenteric ischemia (NOMI), and mesenteric venous occlusion (MVO). Secondary AMI, which is less common, is generally associated with small-bowel obstruction, with additional causes including volvulus, intussusception, and malignancy [1,2]. Computed tomography angiography (CTA) plays a key role in diagnosis. It can simultaneously identify bowel necrosis and vascular abnormalities and distinguish occlusive from non-occlusive AMI [3]. Management of AMI requires a multidisciplinary approach involving general surgery, vascular surgery, interventional radiology, and intensive care specialists. Early diagnosis and prompt initiation of appropriate treatment are critical for improving survival. Nevertheless, acute mesenteric ischemia remains a condition with high mortality despite advances in diagnostic and therapeutic strategies [4].

The clinical and imaging findings of AMI vary depending on factors such as the severity of ischemia and the extent of vascular collateral flow, and they are often nonspecific. This variability makes early diagnosis challenging and may lead to delayed or incorrect identification of the condition, resulting in adverse clinical outcomes [1,2,5]. Therefore, identifying reliable biomarkers for prognostic assessment in AMI patients is crucial in clinical practice. However, no widely accepted or disease-specific prognostic index is currently available for routine clinical use in AMI.

The C-reactive protein–albumin–lymphocyte (CALLY) index is a novel prognostic index calculated using serum albumin, lymphocyte count, and C-reactive protein (CRP), reflecting inflammation, nutritional status, and immune response [6]. Due to its ease of calculation, it is increasingly being used to predict prognosis, and studies have evaluated its prognostic value in various clinical settings, including cardiac ischemia, cerebrovascular disease, and gastrointestinal cancers [6,7].

Reliable prognostic biomarkers for the management of AMI patients remain limited. The prognostic significance of the CALLY index in this patient population has not yet been clearly established. Therefore, the aim of this study was to evaluate the prognostic performance of the CALLY index in patients diagnosed with AMI in the ED.

## 2. Materials and Methods

This study was conducted as a retrospective cohort study. Ethical approval for the study was obtained from the Ethics Committee of Adana City Training and Research Hospital, Health Sciences University, Adana, Turkey on 21 August 2025 (approval number: 16/669). Patients aged 18 years and older who presented to the ED and were diagnosed with AMI between 1 January 2020 and 30 December 2024, were identified through the hospital electronic medical record system and included in the study. Patients who refused treatment, were transferred to another center, had a history of trauma, presented with cardiac arrest on arrival, had chronic liver disease, or were receiving active chemotherapy, radiotherapy, or immunosuppressive therapy were excluded from the study.

The diagnosis of AMI in the ED was established according to the following criteria:

1. Clinical and/or contrast-enhanced CT findings suggestive of bowel injury.
2. Evidence of occlusive or non-occlusive vascular insufficiency in the celiac trunk, superior mesenteric artery, and/or superior mesenteric vein on abdominopelvic CTA.
3. Absence of an alternative diagnosis that could account for the clinical presentation.
4. Surgical findings and/or pathology reports confirming mesenteric ischemia in cases without definitive radiological evidence.

Patients without radiological signs but who underwent surgery due to suspected mesenteric ischemia, and in whom the diagnosis was confirmed surgically or pathologically, were included in the study cohort.

Contrast-enhanced CT findings suggestive of bowel injury included the following:

1. Underlying cause of mesenteric ischemia.
2. Bowel wall necrosis and infarction secondary to mesenteric ischemia.
3. Vascular stasis, thrombosis, and hemorrhage secondary to ischemia.
4. Inflammatory changes associated with or secondary to ischemic processes.
5. Reactive changes such as bowel wall thickening and edema, mesenteric fat stranding, and adjacent free fluid that occur secondary to acute inflammation and ischemia.

The abdominopelvic CTA protocol was performed as follows:

A bolus of 100–150 mL of iodinated contrast (600 mg I/kg) was administered through a cubital vein at a rate of 2.5–4 mL/s. Images were obtained during the arterial phase (25–30 s) and the portal/venous phase (60–70 s). All images were evaluated and reported by radiologists at our institution. Diagnosis was confirmed by surgical findings and/or pathology reports.

Demographic data, including age and sex, comorbidities, vital signs, laboratory results at presentation, imaging findings, and the need for specific interventions in the ED (intubation, blood or blood product transfusion, and administration of inotropic agents) were recorded for all patients. ED outcomes, including surgical intervention, mortality, and admission to the ward or intensive care unit (ICU), were documented. Therapeutic interventions during hospitalization (surgical or conservative) and definitive inpatient outcomes (discharge or death) were systematically recorded. Thirty-day mortality was assessed for all enrolled patients. For patients who underwent surgical treatment, the time from presentation to surgery, the type of procedure performed, and pathology results were documented.

The CALLY index, neutrophil-to-lymphocyte ratio (NLR), platelet-to-lymphocyte ratio (PLR), and CRP-to-lactate ratio were calculated using laboratory values obtained at the time of ED admission for all patients included in the study. The CALLY index was calculated using the following formula [8]:

CALLY index = (Albumin [g/L] × Lymphocyte count [10^9^/L]) ÷ (CRP [mg/L] × 10)

### Statistical Analysis

Data were analyzed using IBM SPSS Statistics for Windows, Version 26.0 (IBM Corp., Armonk, NY, USA). Continuous variables were expressed as mean ± standard deviation (Sd) or median (minimum–maximum), and categorical variables as number and percentage (%).

The normality of continuous variables was assessed using the Kolmogorov–Smirnov test. Comparisons between groups were performed using Student’s *t*-test for normally distributed variables and the Mann–Whitney U test for non-normally distributed variables. Variables found to be significant in the univariate analysis were subsequently included in a multivariate logistic regression model. ROC curve analysis was performed to evaluate the prognostic performance of the CALLY index for mortality. The optimal cut-off value was determined using the Youden index. A *p* < 0.05 was considered statistically significant.

## 3. Results

A total of 111 patients were included in the study. Patients’ ages ranged from 38 to 95 years, with a mean of 69.2 ± 11.8 years. Male patients accounted for 52.3% of the cohort. The most common comorbidities were hypertension (HT) and coronary artery disease (CAD), each present in 45% of patients, whereas chronic kidney disease (CKD) was the least frequent, observed in 7.2% of patients. Comorbid malignancies were observed in 18 patients, most frequently hematologic malignancies (n = 5, 27.8%), followed by genitourinary (n = 4, 22.2%), gastrointestinal (n = 4, 22.2%), pulmonary (n = 3, 16.7%), and intracranial malignancies (n = 2, 11.1%). The demographic and clinical characteristics of all patients with AMI included in the study were shown in Table 1.

The mean length of hospital stay was 17.5 ± 13.1 days. The 30-day mortality rate was 55.9% (n = 62), while mortality within the first 24 h was 17.1% (n = 19). Among patients who died, the mean time to death was 7.6 ± 9.1 days. No deaths occurred after hospital discharge during the 30-day follow-up period. Vital signs and laboratory findings at presentation, categorized by mortality status, were summarized in Table 2, while the distribution of the CALLY index, NLR, PLR, and CRP-to-lactate ratio according to mortality were presented in Table 3. Mechanical ventilation was required in 12.6% of patients, inotropic support in 18.9%, and blood or blood product transfusion in 6.3% of patients in the ED (Table 1).

Among the study population, 34 patients (30.6%) demonstrated CTA findings suggestive of AMI. Other radiological features were as follows: ileus in 50 patients (45%), nonspecific findings in 16 patients (14.4%), free intraperitoneal fluid in 8 patients (7.2%), and evidence of perforation in 3 patients (2.7%). In the 77 (69.4%) patients without CTA findings suggestive of AMI, the diagnosis was confirmed surgically or histopathologically.

All patients were admitted to the ICU and underwent surgical intervention within 24 h of admission. Segmental small intestine resection was performed in 90 patients. Additional resections were performed based on the intraoperative extent of ischemia and necrosis, including the colon in 15 patients, the omentum in 5, the appendix in 2, and combined resections of multiple organs in 4 patients.

The most frequently observed pathological findings were transmural necrosis (80.2%), hemorrhage and congestion (82.9%), and active inflammation (65.8%). Thrombosed vascular structures (53.2%) and submucosal edema (43.2%) were also commonly observed.

The association between various variables and mortality was assessed using univariate analysis. Gender and comorbidity history showed no significant association with mortality (*p* > 0.05; Table 1). The need for inotropic support and mechanical ventilation was significantly higher in the mortality group (*p* < 0.001 for both; Table 1). Among vital signs, systolic and diastolic blood pressure, as well as oxygen saturation, differed significantly between patients who died and survivors (*p* = 0.011, *p* = 0.064, and *p* = 0.023, respectively; Table 2). The Glasgow Coma Score was significantly lower in the mortality group compared to survivors (*p* = 0.002). Laboratory parameters revealed that creatinine, alanine aminotransferase (ALT), C-reactive protein (CRP), and lactate levels were significantly higher in the mortality group (*p* = 0.023, *p* < 0.001, *p* = 0.019, and *p* = 0.004, respectively; Table 2). In contrast, platelet count and albumin levels were significantly lower in the mortality group (*p* = 0.002 and *p* = 0.006, respectively; Table 2). The CALLY index was significantly lower in the mortality group compared to survivors (*p* = 0.011; Table 3). No significant differences were observed between the groups with respect to NLR, PLR, or CRP-to-lactate ratios (*p* > 0.05; Table 3).

Variables that were statistically significant in the univariate analysis were included in the multivariable logistic regression model. In the multivariable logistic regression model which including sex, age, CALLY index, NLR, PLR, CRP-to-lactate ratio, systolic blood pressure, diastolic blood pressure, oxygen saturation, and Glasgow Coma Score, none of the variables were independently associated with mortality (Table 4).

**Figure 1:**
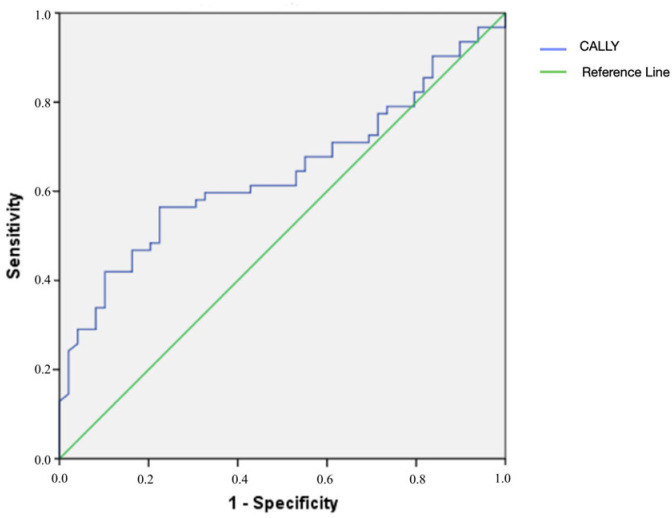
Receiver operating characteristic (ROC) curve analysis of the CALLY index for predicting mortality.

ROC curve analysis demonstrated that the CALLY index had a statistically significant but limited prognostic value in predicting mortality (*p* = 0.011, area under the curve [AUC] = 0.64, 95% confidence interval [CI] = 0.54–0.74; Figure 1). At a cut-off value of 0.0015, the CALLY index showed a sensitivity of 55% and a specificity of 77%.

## 4. Discussion

Despite advances in diagnostic and therapeutic strategies, AMI continues to be associated with persistently high mortality. Due to its nonspecific clinical presentation, diagnosis is often delayed, resulting in rapid clinical deterioration, with reported mortality rates ranging from 60% to 80% in previous studies [1,9]. Although improvements in imaging and surgical techniques have been achieved, overall survival has not substantially improved, underscoring the ongoing challenges in early recognition and effective risk stratification of AMI patients [10,11]. Previous studies reported that AMI predominantly affects elderly individuals, with mean ages ranging from the early 60s to the early 70s, while gender distribution varies across cohorts [10,11,12]. Otto et al. demonstrated that age alone was not an independent predictor of mortality [12], whereas Sumball R. identified age and chronic renal disease as significant risk factors [13]. In a large-scale study, older age, higher comorbidity burden, and male sex were shown to be independently associated with in-hospital mortality, and the need for inotropic support emerged as a strong predictor of poor outcome in critically ill AMI patients [14]. The study population was predominantly elderly, with a mean age of approximately 70 years and a nearly equal gender distribution. Cardiovascular comorbidities, particularly hypertension and coronary artery disease, were frequently observed, supporting the association between AMI and underlying vascular disease. While demographic characteristics and comorbid conditions were not independently associated with mortality in our cohort, the requirement for critical interventions such as intubation, inotropic support, or blood and blood product transfusion was significantly associated with fatal outcomes, highlighting the impact of disease severity at presentation.

The primary goal in AMI management is to minimize the time from symptom onset to diagnosis. Prompt therapeutic intervention is critical, as every 24 h delay doubles mortality [15]. Despite advances in diagnostic and therapeutic approaches, AMI remains a highly lethal condition, and overall survival rates have not significantly improved [10]. In line with this consistently poor prognosis reported in the literature, our study demonstrated a high overall mortality rate of 55.9%.

One of the main challenges in AMI is the delay in establishing a diagnosis and initiating treatment. Therefore, particularly in the emergency department, it is crucial to assess disease progression in individual patients and predict mortality using early diagnostic and prognostic biomarkers. In this context, we evaluated AMI patients and investigated potential risk indices for mortality prediction, including the CALLY index, as well as NLR, PLR, and the CRP-to-lactate ratio.

The CALLY index is calculated using serum albumin, lymphocyte count, and CRP levels. Albumin, synthesized by the liver, reflects protein reserves that are essential during physiological stress. Low preoperative albumin levels are generally associated with higher complication rates and prolonged hospital stays [16]. CRP is an inflammatory marker that can be elevated in acute surgical conditions due to infectious or inflammatory causes. Increased preoperative CRP and low lymphocyte counts have been associated with higher mortality [17,18]. The CALLY index integrates these three parameters—nutrition, immune status, and inflammation—to provide a stronger prognostic prediction. The CALLY index is relatively new in the literature, with some studies demonstrating its prognostic value in specific patient populations. Pan Y. found that the CALLY index was an independent predictor of adverse outcomes in acute ischemic stroke [19]. It has also been suggested as an independent prognostic biomarker for long-term outcomes in patients with colorectal cancer [20]. Han D. et al. reported that an elevated CALLY index was independently associated with cardiovascular mortality over a median follow-up of 122 months [21]. A systematic review by Wu B. et al. concluded that the CALLY index serves as a cost-effective and reliable biomarker for predicting prognosis in digestive system cancers [6]. Zhang J. et al. demonstrated a significant association between the CALLY index and 30-day mortality in critically ill ICU patients [22]. Angın Y. S. et al., in a study of intestinal ischemia in strangulated abdominal wall hernias, reported a mortality rate of 9.6% and suggested that the CALLY index could be valuable in predicting intestinal ischemia and guiding surgical decisions [23]. In our study, patients were critically ill with high baseline mortality and required surgery, which may explain the lower performance of the CALLY index. In our study, while the three parameters were evaluated individually, albumin and CRP were significant predictors of mortality in univariate analysis, whereas lymphocyte count was not. In the multivariate analysis, however, CRP and albumin lost their statistical significance, and the CALLY index was also not an independent predictor. When CALLY index values were assessed, patients who died had significantly lower values than survivors. ROC analysis demonstrated that the CALLY index had a modest but statistically significant ability to predict 30-day mortality in patients with AMI (AUC = 0.64, 95% CI: 0.54–0.74). Given its relatively low sensitivity, the CALLY index alone may not be sufficient for clinical decision-making in emergency settings. Strong associations between mortality and interventions such as intubation and vasopressor use in our study indicate that clinical severity may play a larger role than laboratory-based indices. Nevertheless, the CALLY index can provide additional prognostic information beyond standard clinical assessments, complementing established clinical and laboratory markers.

In the literature, various laboratory parameters have been investigated for both diagnostic and prognostic purposes in AMI. Destek et al. reported that preoperative CRP levels were effective biomarkers for diagnosing AMI, differentiating its subtypes, and assessing the clinical course; however, markers such as L-lactate, D-dimer, leukocyte count, and NLR did not demonstrate predictive value across all AMI subtypes [24]. In contrast, Aktimur et al. suggested that a high NLR value (>9.9) could serve as a significant diagnostic marker for AMI [25]. Kaçer et al. found that the CRP/albumin ratio was a strong predictor of in-hospital mortality in AMI patients, outperforming leukocyte count, NLR, and lactate [26]. Conversely, Vural and Özozan reported that preoperative NLR and PLR could not independently predict 30-day mortality in surgically treated acute intestinal ischemia patients [27]. Uludağ et al. demonstrated that NLR and serum albumin levels might be useful for assessing AMI severity [10]. In our study, however, none of these parameters—including NLR, PLR, and the CRP-to-lactate ratio—showed a significant association with mortality, suggesting that their prognostic utility may be limited in critically ill AMI patients.

Our study was limited by its retrospective, single-center design, which may restrict the generalizability of the results, particularly as only surgically treated, critically ill ICU patients were included. In addition, the small sample size and the inability to assess certain prognostic factors, such as perioperative and postoperative hemodynamic status and repeated laboratory measurements, represent further limitations. A detailed etiological classification (arterial embolism, arterial thrombosis, non-occlusive mesenteric ischemia, venous thrombosis, or secondary causes) could not be reliably established for all patients, mainly due to the retrospective design, heterogeneous imaging findings at presentation, and the fact that a substantial proportion of AMI diagnoses were confirmed intraoperatively or histopathologically. Finally, residual confounding cannot be excluded, and the results have not been externally validated, which may further limit the generalizability of our findings.

## 5. Conclusions

In patients with AMI, the CALLY index was significantly associated with mortality in univariate analysis, but it was not an independent predictor in multivariate analysis. Nevertheless, it may offer additional prognostic information when considered alongside other clinical and laboratory parameters.

## Figures and Tables

**Table 1:** Analysis of 30-day mortality according to demographic and clinical characteristics in patients with acute mesenteric ischemia.

Variables (n = 111)		Mortality (+) (n = 62)		Mortality (−) (n = 49)		Total (n = 111)		*p* Value
		n	%	n	%	n	%	
Gender	Female	32	60.4	21	39.6	53	47.7	0.359
	Male	30	51.7	28	48.3	58	52.3	
Comorbidities								
Diabetes mellitus	Yes	23	57.5	17	42.5	40	36	0.793
	No	39	54.9	32	45.1	71	64	
Hypertension	Yes	29	58	21	42	50	45	0.680
	No	33	54.1	28	45.9	61	55	
Cerebrovascular event	Yes	29	58	21	42	13	11.7	0.680
	No	33	54.1	28	45.9	98	88.3	
Coronary artery disease	Yes	10	55.6	8	44.4	50	45	0.978
	No	52	55.9	41	44.1	61	55	
Malignancy	Yes	5	38.5	8	61.5	18	16.2	0.179
	No	57	58.2	41	41.8	93	83.8	
Chronic renal failure	Yes	5	62.5	3	37.5	8	7.2	1.000
	No	57	55.3	46	44.7	103	92.8	
Arrhythmia	Yes	16	55.2	13	44.8	29	26.1	0.931
	No	46	56.1	36	43.9	82	73.9	
Congestive heart failure	Yes	3	33.3	6	66.7	9	8.1	0.180
	No	59	57.8	43	42.2	102	91.9	
Treatment in the emergency department								
Mechanical ventilation support	Yes	14	100	0	0	14	12.6	<0.001
	No	48	49.5	49	50.5	97	87.4	
Inotropic agent support	Yes	19	9.5	2	90.5	21	18.9	<0.001
	No	43	47.8	47	52.2	90	81.1	
Blood and blood product replacement	Yes	4	57.1	3	42.9	7	6.3	1.000
	No	58	55.8	46	44.2	104	93.7	

Sd: Standard deviation.

**Table 2:** Analysis of 30-day mortality according to vital signs and laboratory parameters in patients with acute mesenteric ischemia.

Vital Signs and Laboratory Parameters	Mortality (+) (n = 62) Mean ± Sd	Mortality (−) (n = 49) Mean ± Sd	Total (n = 111) Mean ± Sd	*p* Value
Body temperature	36.6 ± 0.4	36.6 ± 0.5	36.6 ± 0.5	0.489 **
Heart rate (beats/min)	106.6 ± 20.5	100.6 ± 20.4	103.9 ± 20.6	0.125
Systolic blood pressure (mmHg)	111.7 ± 31.7	125.0 ± 22.7	117.5 ± 28.7	0.011
Diastolic blood pressure (mmHg)	67.0 ± 20.3	76.1 ± 11.7	71.0 ± 17.6	0.004
Oxygen saturation (%)	95.9 ± 3.1	97.1 ± 2.2	96.4 ± 2.7	0.023
Glasgow coma score	14.2 ± 2.7	15.0 ± 0.0	14.5 ± 2.1	0.002 **
Leukocyte(10^3^/µL)	18.0 ± 11.1	17.5 ± 8.9	17.8 ± 10.2	0.798 **
Hemoglobin (g/dL)	12.7 ± 2.7	13.2 ± 2.5	12.9 ± 2.6	0.305
Hematocrit (%)	38.3 ± 7.7	39.5 ± 7.2	38.8 ± 7.5	0.396
Platelet (×10^3^/µL)	235.9 ± 99	303.8 ± 11.9	265.9 ±1133.6	0.002 **
Neutrophil count (×10^3^/µL)	15.4 ± 10.1	15.2 ± 8.5	15.1 ± 9.6	0.671 **
Lymphocyte count (×10^3^/µL)	1.31 ± 2.2	1.07 ± 7.92	1.5 ± 1.9	0.526 **
Glucose (mg/dL)	181.8 ± 89.8	181.2 ± 75	181.5 ± 83.2	0.971
Urea (mg/dL)	82.5 ± 52.4	67.7 ± 42.5	76.0 ± 48.6	0.082 **
Creatinine (mg/dL)	2.2 ± 1.8	1.4 ± 0.9	1.9 ± 1.5	0.023 **
Albumin (g/L)	29.7 ± 8.1	33.7 ± 7.1	31.4 ± 7.9	0.006
Total protein (g/L)	57.2 ± 13.6	61.5 ± 10.7	59.1 ± 12.5	0.061
CRP (mg/L)	235.3 ± 166.8	164.2 ± 148.3	203.9 ± 162.2	0.019
Sodium (mmol/L)	136.3 ± 5.4	136.4 ± 3.8	136.3 ± 4.7	0.860
Potassium (mmol/L)	4.5 ± 1.0	4.3 ± 0.8	4.4 ± 0.9	0.376
Calcium (mg/dL)	8.7 ± 1.0	8.9 ± 0.9	8.8 ± 1.0	0.347
ALT (U/L)	115.2 ± 417.8	19.4 ± 11.6	72.9 ± 31.9	<0.001 **
Lactate	56.9 ± 44.1	35.1 ± 22.8	47.3 ± 37.7	0.004 **

*t* test, ** Mann–Whitney U test; dL: deciliter; g: gram; L: liter; mg: milligram; mmHg: millimeters of mercury; mmol: millimoles; min: minute; µL: microliter; pCO_2_: Partial carbon dioxide; U: unit.

**Table 3:** Association between the CALLY index, NLR, PLR, CRP-to-lactate ratio, and 30-day mortality.

	Mortality (+) (n = 62)		Mortality (−) (n = 49)		Total (n = 111)		*p* Value
	Mean ± Sd	Medyan (min–max)	Mean ± Sd	Medyan (min–max)	Mean ± Sd	Medyan (min–max)	
CALLY	0.30 ± 1.28	0.01 (0.01–7.17)	0.36 ± 1.32	0.02 (0.01–6.80)	0.32 ± 1.28	0.02 (0.01–7.17)	**0.011**
NLR	21.43 ± 16.63	16.63 (1.13–67.00)	22.23 ± 21.61	14.58 (1.18–105.67)	21.78 ± 18.90	15.33 (1.13–105.67)	0.713
PLR	347.80 ± 293.25	294.02 (14.88–1640.0)	429.80 ± 373.86	321.67 (48.86–1645.00)	384.00 ± 332.20	306.00 (14.88–1645.00)	0.199
CRP/Laktat	8.26 ± 10.87	4.48 (0.03–58.67)	7.36 ± 9.01	4.56 (0.03–36.10)	7.86 ± 10.06	4.53 (0.03–58.67)	0.771

Mann–Whitney U test; CRP: C-reactive protein; min: Minimum; max: Maximum; NLR: Neutrophil/lymphocyte ratio; PLR: Platelet/lymphocyte ratio.

**Table 4:** Multivariate logistic regression analysis of factors associated with 30-day mortality in patients with acute mesenteric ischemia.

Variables (n = 111)	OR	*p* Value	%95 CI
Gender	1.55	0.338	0.63–3.82
Age	1.01	0.782	0.97–1.05
CALLY	1.05	0.773	0.76–1.46
NLR	1.02	0.278	0.99–1.05
PLR	1.00	0.152	1.00–1.00
CRP/Laktat	1.01	0.722	0.96–1.05
Systolic blood pressure	1.00	0.809	0.97–1.02
Diastolic blood pressure	0.99	0.565	0.94–1.03
Oxygen saturation	0.86	0.116	0.72–1.04
Glasgow coma score	0.01	0.996	0.00–0.00
Constant B = 280.015; *p* = 0.996; Exp(B) = 4.063; Nagelkerke R Square = 0.257			

CI: Confidence interval; OR: Odds ratio.

## Data Availability

The datasets used and/or analysed during the current study are available from the corresponding author on reasonable request.

## Abbreviations

AMI	Acute mesenteric ischemia
AUC	Area under the curve
CALLY	The C-reactive protein–albumin–lymphocyte
CI	Confidence Interval
CRP	C-reactive protein
CTA	Computed tomography angiography
ED	Emergency department
ICU	Intensive care unit
MAO	Mesenteric arterial occlusion
MVO	Mesenteric venous occlusion
NLR	Neutrophil-to-lymphocyte ratio
NOMI	Non-occlusive mesenteric ischemia
PLR	Platelet-to-lymphocyte ratio
ROC	Receiver Operating Characteristic
